# Crystal structure and Hirshfeld surface analysis of diethyl 5-(2-cyano­phen­oxy)isophthalate

**DOI:** 10.1107/S2056989020004508

**Published:** 2020-05-22

**Authors:** Mohd Muslim, Arif Ali, Saima Kamaal, Musheer Ahmad, Mohd Afzal, Maksym O. Plutenko

**Affiliations:** a Department of Applied Chemistry, ZHCET, Aligarh Muslim University, Aligarh, 202002, (UP), India; b Catalytic Chemistry Research Chair, Department of Chemistry, College of Science, KSU, Riyadh 11451, Saudi Arabia; c Department of Chemistry, National Taras Shevchenko University, Volodymyrska Street 64, 01601 Kyiv, Ukraine

**Keywords:** crystal structure, 5-hy­droxy-isophthalic acid, hydrogen bonding, 2-fluoro­benzo­nitrile

## Abstract

The title compound, C_19_H_17_NO_5_, obtained by ether bond formation between the reagents, crystallizes in the monoclinic space group *P*2_1_/*c*. The compound is non-planar, subtending a dihedral angle of 82.38 (4)° between the plane of hy­droxy isophthalate-based ester and that of the benzo­nitrile moiety. The mol­ecule is bent at the ether linkage, with a C_ar­yl_—O—C_ar­yl_ bond angle of 116.74 (11)°. In the crystal, mol­ecules are linked by C—H⋯O hydrogen bonds and other weak inter­actions forming a supra­molecular framework. A Hirshfeld surface analysis was performed to generate two-dimensional fingerprint plots, which reveal the type of inter­actions occurring in the vicinity of the mol­ecule.

## Abstract 2

The title compound is non-planar, subtending a dihedral angle of 82.38 (4)° between the plane of hy­droxy isophthalate-based ester and that of the benzo­nitrile moiety. The mol­ecule is bent at the ether linkage, with a C_ar­yl_—O—C_ar­yl_ bond angle of 116.74 (11)°. In the crystal, mol­ecules are linked by C—H⋯O hydrogen bonds and other weak inter­actions forming a supra­molecular framework.

## Chemical context   

5-Hy­droxy­isophthalic acid and its derivatives have been used in the synthesis of several organic ligands. This type of ligand has an isophthalate moiety, which has oxygen-rich carbon chains that are sufficiently reactive to incorporate functionality, followed by conjugation with biomolecular compounds (Calderon *et al.*, 2010 ▸; Khandare *et al.*, 2012 ▸). Carboxyl­ate-containing ligands have been used for the synthesis of coordination polymers because of their flexible nature. The flexibility of the ligand and hardness of metal ions improve the stability of coordination polymers (Ahmad *et al.*, 2012 ▸). Coordination polymers have been used in various types of applications as a result of their physical properties, which include ferromagnetic behaviour, anti­ferromagnetic ordering, spin canting and metamagnetism (Wang *et al.*, 2005 ▸; Liu *et al.*, 2010 ▸). Several types of framework have been obtained, such as metal complexes, clusters, and metal–organic frameworks by linking of the flexible organic linker and metal ion, leading to inter­esting magnetic properties (Cheon & Suh, 2009 ▸; Wang *et al.*, 2009 ▸). Organic ligands containing ether linkages have been used to synthesize magnetic materials because these types of organic ligands exhibit a binding ability that can efficiently transmit magnetic coupling (Coronado *et al.*, 2000 ▸; Masciocchi *et al.*, 2009 ▸; Yu *et al.*, 2010 ▸).
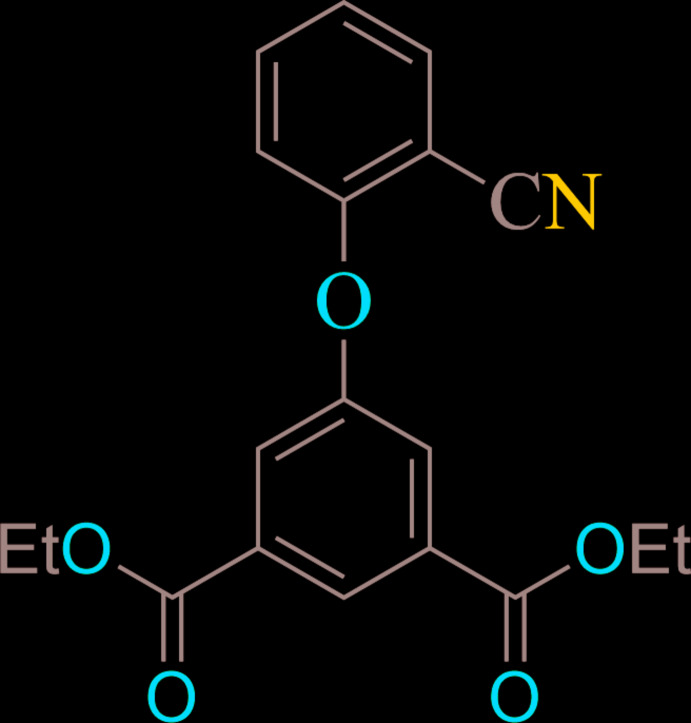


## Structural commentary   

**Figure 1:**
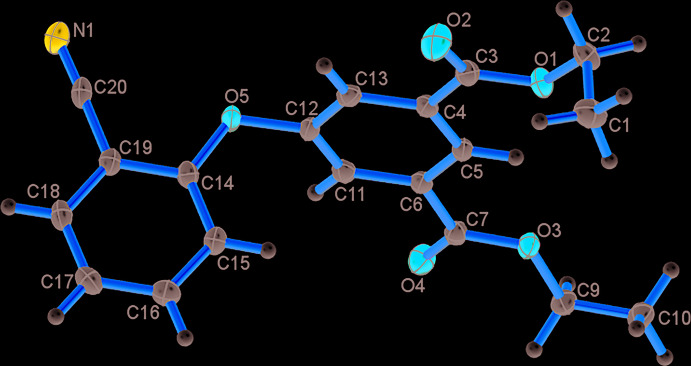
The mol­ecular structure of the title compound, with atom labelling. Displacement ellipsoids are drawn at the 50% level.

The mol­ecular structure of the title compound is shown in Fig. 1 ▸. The compound crystallizes in the monoclinic space group *P*2_1_/*c*. The asymmetric unit contains one unit of 5-hy­droxy-isophthalic acid diethyl ester and one unit of benzo­nitrile, connected by an ether bridge linkage. The mol­ecule is non-planar, with a C12—O5—C14 bond angle of 116.74 (11)° at the ether group, and a C14—O5—C12—C13 torsion angle at the bridge of −97.37 (2)°. The C12—O5 bond length, 1.4025 (17) Å, is comparable to the C-O bond lengths obtained for similar ligands. The C3—O1 and C3—O2 bond lengths are 1.3377 (18) and 1.2061 (19) Å, respectively, and are in the expected ranges (Cambridge Structural Database; Groom *et al.*, 2016 ▸).

## Supra­molecular features   

**Figure 2:**
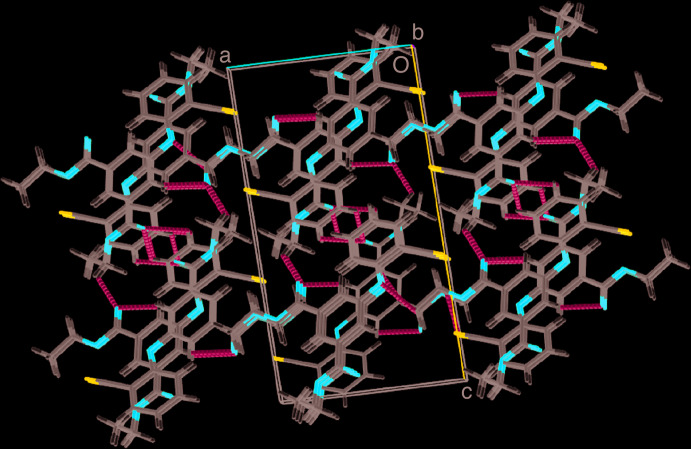
A view along the *b* axis of the crystal packing of the title compound. Hydrogen bonds are shown as dashed lines.

**Figure 3:**
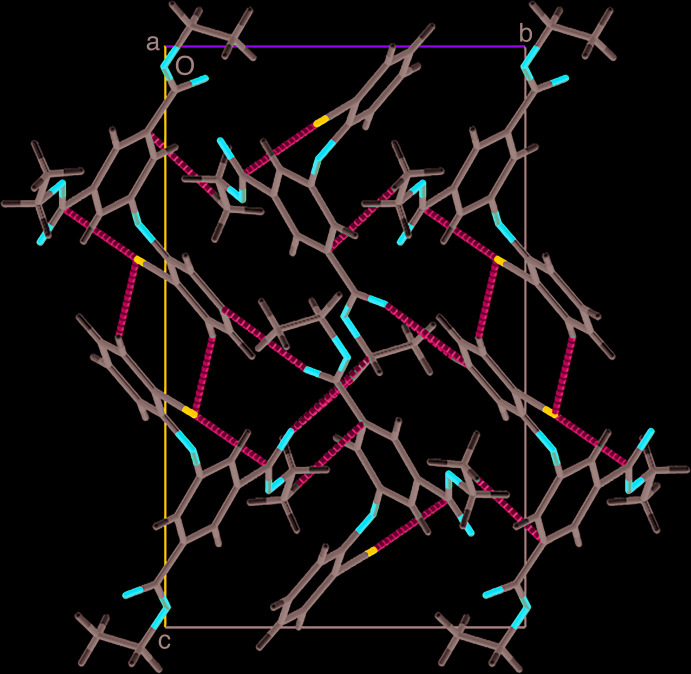
A view along the *a* axis of the crystal packing of the title compound. The C—H⋯π and other weak inter­actions are indicated by dashed lines.

In the crystal, the mol­ecules are connected through C2—H2A⋯O4, C16—H16⋯O2 and C13—H13⋯O4 hydrogen bonds (Table 1 ▸, Fig. 2 ▸). They are linked by a series of C10—H10*C*⋯π and C3—O2⋯C16, C7—O4⋯C2 and C20—N1⋯C7 weak interactions, forming an extended supra­molecular framework (Fig. 3 ▸). π–π interactions with *Cg*1⋯*Cg*2(1 − *x*, 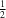 + *y*, 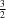 − *z*) = 3.9572 (9) Å where *Cg*1 and*Cg*2 are the centroids of the C4–C6/C11–C13 and C14–C19 rings, respectively, and a C—H⋯N inter­action are also observed.

## Hirshfeld analysis   

**Figure 4:**
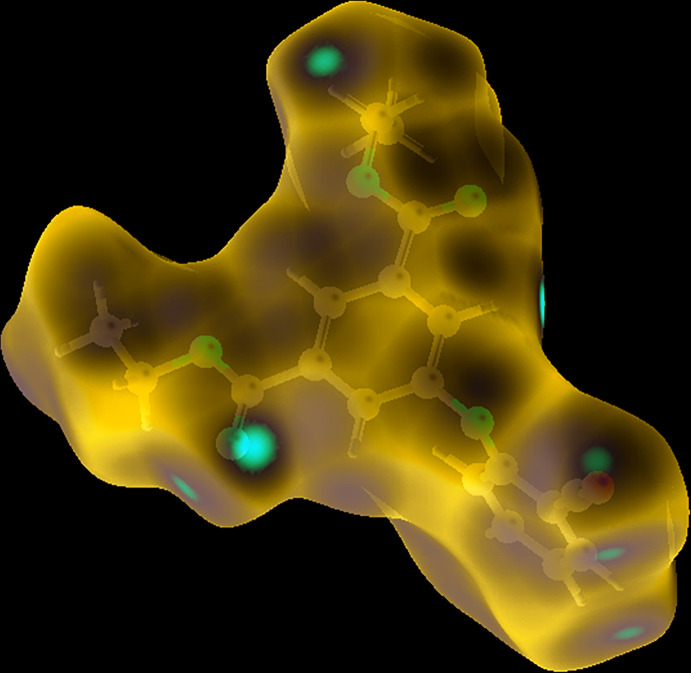
The Hirshfeld surface of the crystal structure mapped over *d*_norm_, in the colour range −0.174 to 1.315 a.u..

**Figure 5:**
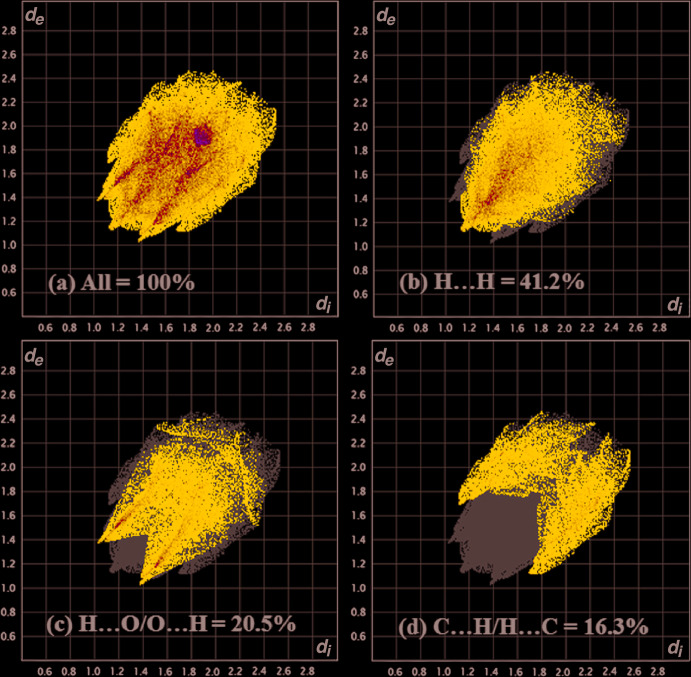
(*a*) A full 2D fingerprint plot of the title compound, and delineated into (*b*) H⋯H (41.2%) (*c*) H⋯O/O⋯H (20.5%) and (*d*) C⋯H/H⋯C (16.3%) contacts, which are the major inter­actions present in the crystal structure.

The Hirshfeld surface analysis (Spackman & Jayatilaka, 2009 ▸) and the associated two-dimensional fingerprint plots (McKinnon, *et al.*, 2007 ▸) were performed with *Crystal Explorer17* (Turner *et al.*, 2017 ▸) to investigate the inter­molecular inter­actions and surface morphology of the crystal structure. The Hirshfeld surface mapped over *d*_norm_ (Fig. 4 ▸) in the colour range −0.174 to 1.315 a.u. from red (shorter distance than the sum of van der Waals radii) and white to blue (longer distance than the sum of van der Waals radii). The bright red spot on the *d*_norm_ surface corresponds to a weak inter­action *e.g*. hydrogen bonding, blue indicates close contacts and a white spot shows van der Waals inter­actions. In the crystal there are three major types of inter­action (H⋯H = 41.2%, H⋯O = 20.5%, C⋯H = 16.3%) on the *d*_norm_ surface. The two-dimensional fingerprint plots are shown in Fig. 5 ▸. The inter­action order of *d*_norm_ on the 2D fingerprint plot (H⋯H)>(H⋯O)>(C⋯H) represents the nature of the packing in the crystal structure. The contribution of these major inter­actions (H⋯H, O⋯H/H⋯O, and C⋯H/H⋯C), governs the overall packing of crystal structure.

## Database survey   

A search of the Cambridge Structural Database (CSD version 5.39, update of May 2018; Groom *et al.*, 2016 ▸) for 5-hy­droxy-isophthalic acid derivatives gave 38 hits for structures that include atomic coordinates. In most of the derivatives, the phenolic group is replaced by an alk­oxy, a substituted alk­oxy or a substituted phen­oxy moiety. Only in three of the 5-hy­droxy-isophthalic acid derivatives is the carboxyl group modified: IDIYIE (Petek *et al.*, 2006 ▸), NUHTAM (Feng *et al.*, 2009 ▸), EVIBOB (Yang *et al.*, 2011 ▸). In all these compounds, the hydroxyl groups of the carboxyl moieties have been replaced by meth­oxy groups and the phenolic group is replaced by a substituted alk­oxy or a substituted phen­oxy moiety.

## Synthesis and crystallization   

5-Hy­droxy­isophthalic acid diethyl ester (3.7 g, 14.9 mmol) was mixed with dried K_2_CO_3_ (3.3g, 22.3 mmol) in a 100 ml round-bottom flask under an inert atmosphere and then treated with dry DMF (20 ml) . The mixture was stirred for 30 minutes at 353 K followed by addition of 2-fluoro-benzo­nitrile (1.8 ml, 16.6 mmol) and the resulting mixture was stirred for 24 h in an oil-bath at 353 K. After this period, the solution was allowed to cool to room temperature and then poured into ice-cold water (100 ml) with vigorous stirring, to afford a white precipitate, which was collected by filtration, washed with water, and dried under vacuum. Yield: 4.6 g (90%). Melting point 325 K. The ligand was crystallized from a solution in ethanol, the resultant solution was filtered and kept for slow evaporation. After 2–3 weeks, block-shaped colourless crystal were obtained, which were suitable for single-crystal X-ray diffraction analysis.

## Refinement   

Crytal data, data collection and structure refinement details are summarized in Table 2 ▸. The H atoms were freely refined.

## Supplementary Material

Crystal structure: contains datablock(s) global, I. DOI: 10.1107/S2056989020004508/ex2031sup1.cif

Structure factors: contains datablock(s) I. DOI: 10.1107/S2056989020004508/ex2031Isup2.hkl

Supporting information file. DOI: 10.1107/S2056989020004508/ex2031Isup3.cml

CCDC reference: 1981835

Additional supporting information:  crystallographic information; 3D view; checkCIF report

## Figures and Tables

**Table 1:** Hydrogen-bond geometry (Å, °) *Cg*1 is the centroid of the C4–C6/C11–C13 ring.

*D*—H⋯*A*	*D*—H	H⋯*A*	*D*⋯*A*	*D*—H⋯*A*
C2—H2B⋯O4^i^	0.98 (2)	2.50 (2)	3.2071 (2)	128 (1)
C13—H13⋯O4^ii^	0.936 (16)	2.514 (16)	3.4179 (2)	163.5 (12)
C16—H16⋯O2^iii^	0.997 (17)	2.516 (17)	3.1941 (2)	125.7 (13)
C18—H18⋯N1^iv^	0.945 (17)	2.629 (17)	3.430 (2)	143.0 (13)
C10—H10C⋯*Cg*1^v^	1.00 (2)	2.96 (2)	3.7893 (19)	140 (2)

Symmetry codes: (i) 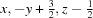; (ii) 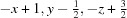; (iii) 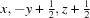; (iv) 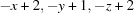; (v) 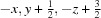.

**Table 2:** Experimental details

Crystal data	
Chemical formula	C_19_H_17_NO_5_
*M*_r_	339.35
Crystal system, space group	Monoclinic, *P*2_1_/*c*
Temperature (K)	100
*a*, *b*, *c* (Å)	9.3581 (5), 10.5306 (6), 17.0141 (10)
β (°)	91.967 (2)
*V* (Å^3^)	1675.69 (16)
*Z*	4
Radiation type	Mo *K*α
μ (mm^−1^)	0.10
Crystal size (mm)	0.39 × 0.27 × 0.16

Data collection	
Diffractometer	Bruker APEXII CCD
Absorption correction	Multi-scan (*SADABS*; Bruker, 2016 ▸)
*T*_min_, *T*_max_	0.611, 0.746
No. of measured, independent and observed [*I* ≥ 2σ(*I*)] reflections	25974, 4149, 3134
*R*_int_	0.062
(sin θ/λ)_max_ (Å^−1^)	1.174

Refinement	
*R*[*F* ^2^ > 2σ(*F* ^2^)], *wR*(*F* ^2^), *S*	0.044, 0.110, 1.10
No. of reflections	4149
No. of parameters	294
H-atom treatment	All H-atom parameters refined
Δρ_max_, Δρ_min_ (e Å^−3^)	0.30, −0.31

Computer programs: *APEX2* and *SAINT* (Bruker, 2016 ▸), *olex2.solve* and *olex2.refine* (Bourhis *et al.*, 2015 ▸) and *OLEX2* (Dolomanov *et al.*, 2009 ▸).

## supplementary crystallographic information

### Crystal data

**Table:**

C_19_H_17_NO_5_	*F*(000) = 712.4237
*M**_r_* = 339.35	*D*_x_ = 1.345 Mg m^−^^3^
Monoclinic, *P*2_1_/*c*	Mo *K*α radiation, λ = 0.71073 Å
*a* = 9.3581 (5) Å	Cell parameters from 7552 reflections
*b* = 10.5306 (6) Å	θ = 3.2–28.2°
*c* = 17.0141 (10) Å	µ = 0.10 mm^−^^1^
β = 91.967 (2)°	*T* = 100 K
*V* = 1675.69 (16) Å^3^	Block, colourless
*Z* = 4	0.39 × 0.27 × 0.16 mm

### Data collection

**Table:**

Bruker APEXII CCD diffractometer	3134 reflections with *I*≥ 2σ(*I*)
φ and ω scans	*R*_int_ = 0.062
Absorption correction: multi-scan (SADABS; Bruker, 2016)	θ_max_ = 56.6°, θ_min_ = 5.8°
*T*_min_ = 0.611, *T*_max_ = 0.746	*h* = −12→12
25974 measured reflections	*k* = −14→14
4149 independent reflections	*l* = −22→22

### Refinement

**Table:**

Refinement on *F*^2^	0 constraints
Least-squares matrix: full	Primary atom site location: iterative
*R*[*F*^2^ > 2σ(*F*^2^)] = 0.044	All H-atom parameters refined
*wR*(*F*^2^) = 0.110	*w* = 1/[σ^2^(*F*_o_^2^) + (0.035*P*)^2^ + 0.7212*P*] where *P* = (*F*_o_^2^ + 2*F*_c_^2^)/3
*S* = 1.10	(Δ/σ)_max_ = 0.001
4149 reflections	Δρ_max_ = 0.30 e Å^−^^3^
294 parameters	Δρ_min_ = −0.31 e Å^−^^3^
0 restraints	

### Fractional atomic coordinates and isotropic or equivalent isotropic displacement parameters (Å^2^)

**Table:**

	*x*	*y*	*z*	*U*_iso_*/*U*_eq_	
O1	0.22713 (11)	0.50496 (10)	0.53544 (6)	0.0229 (2)	
O2	0.42747 (13)	0.38970 (12)	0.55579 (7)	0.0360 (3)	
O3	0.06230 (11)	0.78631 (10)	0.73574 (6)	0.0213 (2)	
O4	0.20643 (11)	0.84802 (11)	0.83720 (7)	0.0266 (3)	
O5	0.64745 (11)	0.57614 (10)	0.80523 (6)	0.0232 (2)	
N1	1.00269 (15)	0.58018 (13)	0.86616 (8)	0.0285 (3)	
C1	0.1112 (2)	0.31216 (17)	0.48736 (11)	0.0312 (4)	
H1a	0.026 (2)	0.3334 (18)	0.5175 (11)	0.036 (5)*	
H1b	0.174 (2)	0.255 (2)	0.5194 (12)	0.044 (6)*	
H1c	0.079 (2)	0.265 (2)	0.4393 (12)	0.045 (6)*	
C2	0.19053 (19)	0.42986 (16)	0.46556 (9)	0.0255 (3)	
H2a	0.279 (2)	0.4097 (17)	0.4384 (11)	0.030 (5)*	
H2b	0.130 (2)	0.4875 (19)	0.4334 (11)	0.036 (5)*	
C3	0.34773 (16)	0.47318 (14)	0.57513 (9)	0.0216 (3)	
C4	0.37168 (16)	0.55112 (13)	0.64759 (9)	0.0187 (3)	
C5	0.26900 (15)	0.63480 (14)	0.67416 (9)	0.0180 (3)	
H5	0.1801 (18)	0.6472 (16)	0.6445 (10)	0.020 (4)*	
C6	0.29409 (15)	0.70006 (13)	0.74438 (9)	0.0184 (3)	
C7	0.18526 (15)	0.78627 (14)	0.77797 (9)	0.0193 (3)	
C9	−0.05528 (16)	0.85475 (16)	0.77059 (10)	0.0238 (3)	
H9a	−0.0642 (18)	0.8230 (17)	0.8257 (11)	0.026 (4)*	
H9b	−0.0293 (18)	0.9469 (17)	0.7713 (10)	0.024 (4)*	
C10	−0.18649 (17)	0.82704 (18)	0.72010 (11)	0.0282 (4)	
H10a	−0.1759 (19)	0.8645 (18)	0.6671 (12)	0.033 (5)*	
H10b	−0.202 (2)	0.735 (2)	0.7152 (11)	0.035 (5)*	
H10c	−0.271 (2)	0.865 (2)	0.7460 (12)	0.044 (6)*	
C11	0.42139 (16)	0.68242 (14)	0.78809 (9)	0.0198 (3)	
H11	0.4401 (17)	0.7255 (16)	0.8374 (10)	0.021 (4)*	
C12	0.52017 (15)	0.59670 (14)	0.76114 (9)	0.0193 (3)	
C13	0.49852 (16)	0.53133 (14)	0.69155 (9)	0.0199 (3)	
H13	0.5670 (17)	0.4739 (16)	0.6743 (9)	0.017 (4)*	
C14	0.64157 (15)	0.49105 (14)	0.86643 (9)	0.0189 (3)	
C15	0.51899 (17)	0.42882 (14)	0.88849 (9)	0.0216 (3)	
H15	0.429 (2)	0.4474 (18)	0.8609 (11)	0.034 (5)*	
C16	0.52654 (18)	0.34329 (15)	0.95050 (9)	0.0243 (3)	
H16	0.4388 (18)	0.2977 (16)	0.9645 (10)	0.025 (4)*	
C17	0.65468 (18)	0.31972 (15)	0.99145 (9)	0.0254 (3)	
H17	0.659 (2)	0.2606 (18)	1.0337 (11)	0.034 (5)*	
C18	0.77621 (17)	0.38328 (15)	0.97039 (9)	0.0229 (3)	
H18	0.8631 (18)	0.3700 (16)	0.9991 (10)	0.023 (4)*	
C19	0.77115 (15)	0.46874 (14)	0.90750 (9)	0.0194 (3)	
C20	0.89888 (16)	0.53164 (15)	0.88389 (9)	0.0218 (3)	

### Atomic displacement parameters (Å^2^)

**Table:**

	*U*^11^	*U*^22^	*U*^33^	*U*^12^	*U*^13^	*U*^23^
O1	0.0265 (6)	0.0223 (5)	0.0195 (5)	0.0021 (4)	−0.0050 (4)	−0.0020 (4)
O2	0.0327 (7)	0.0383 (7)	0.0368 (7)	0.0128 (6)	−0.0025 (5)	−0.0149 (6)
O3	0.0165 (5)	0.0264 (6)	0.0208 (5)	0.0056 (4)	−0.0010 (4)	−0.0018 (4)
O4	0.0237 (6)	0.0285 (6)	0.0274 (6)	−0.0002 (5)	−0.0019 (5)	−0.0096 (5)
O5	0.0154 (5)	0.0285 (6)	0.0252 (6)	−0.0019 (4)	−0.0048 (4)	0.0098 (5)
N1	0.0227 (7)	0.0311 (7)	0.0313 (8)	−0.0018 (6)	−0.0054 (6)	−0.0021 (6)
C1	0.0372 (10)	0.0299 (9)	0.0265 (9)	−0.0063 (8)	0.0035 (8)	−0.0051 (7)
C2	0.0340 (9)	0.0252 (8)	0.0172 (7)	−0.0020 (7)	−0.0022 (7)	−0.0022 (6)
C3	0.0219 (8)	0.0212 (7)	0.0218 (8)	0.0022 (6)	0.0006 (6)	0.0015 (6)
C4	0.0186 (7)	0.0183 (7)	0.0191 (7)	−0.0001 (6)	0.0005 (6)	0.0027 (6)
C5	0.0156 (7)	0.0185 (7)	0.0199 (7)	−0.0004 (6)	−0.0015 (6)	0.0039 (6)
C6	0.0173 (7)	0.0179 (7)	0.0200 (7)	−0.0004 (5)	0.0002 (6)	0.0036 (6)
C7	0.0174 (7)	0.0194 (7)	0.0212 (7)	−0.0007 (6)	−0.0006 (6)	0.0008 (6)
C9	0.0203 (8)	0.0280 (8)	0.0232 (8)	0.0073 (6)	0.0019 (6)	−0.0025 (7)
C10	0.0185 (8)	0.0373 (10)	0.0288 (9)	0.0051 (7)	0.0011 (7)	−0.0033 (8)
C11	0.0196 (7)	0.0212 (7)	0.0185 (7)	−0.0034 (6)	−0.0012 (6)	0.0024 (6)
C12	0.0139 (7)	0.0218 (7)	0.0220 (8)	−0.0019 (6)	−0.0026 (6)	0.0070 (6)
C13	0.0180 (7)	0.0192 (7)	0.0226 (8)	0.0017 (6)	0.0034 (6)	0.0048 (6)
C14	0.0192 (7)	0.0170 (7)	0.0204 (7)	0.0020 (6)	−0.0029 (6)	0.0001 (6)
C15	0.0192 (7)	0.0229 (7)	0.0225 (8)	0.0002 (6)	−0.0024 (6)	0.0001 (6)
C16	0.0274 (8)	0.0217 (7)	0.0241 (8)	−0.0002 (6)	0.0025 (6)	0.0009 (6)
C17	0.0334 (9)	0.0241 (8)	0.0187 (8)	0.0049 (7)	−0.0005 (6)	0.0024 (6)
C18	0.0245 (8)	0.0252 (8)	0.0184 (7)	0.0060 (6)	−0.0058 (6)	−0.0033 (6)
C19	0.0185 (7)	0.0197 (7)	0.0199 (7)	0.0018 (6)	−0.0024 (6)	−0.0041 (6)
C20	0.0194 (8)	0.0232 (7)	0.0222 (8)	0.0032 (6)	−0.0072 (6)	−0.0029 (6)

### Geometric parameters (Å, º)

**Table:**

O1—C2	1.4588 (18)	C9—H9a	1.002 (18)
O1—C3	1.3377 (18)	C9—H9b	1.001 (17)
O2—C3	1.2061 (19)	C9—C10	1.503 (2)
O3—C7	1.3355 (17)	C10—H10a	0.992 (19)
O3—C9	1.4583 (17)	C10—H10b	0.99 (2)
O4—C7	1.2100 (18)	C10—H10c	1.00 (2)
O5—C12	1.4025 (17)	C11—H11	0.964 (17)
O5—C14	1.3763 (18)	C11—C12	1.382 (2)
N1—C20	1.147 (2)	C12—C13	1.379 (2)
C1—H1a	0.99 (2)	C13—H13	0.936 (17)
C1—H1b	0.99 (2)	C14—C15	1.384 (2)
C1—H1c	1.00 (2)	C14—C19	1.399 (2)
C1—C2	1.498 (2)	C15—H15	0.973 (19)
C2—H2a	0.988 (19)	C15—C16	1.387 (2)
C2—H2b	0.98 (2)	C16—H16	0.988 (17)
C3—C4	1.492 (2)	C16—C17	1.388 (2)
C4—C5	1.391 (2)	C17—H17	0.950 (19)
C4—C13	1.397 (2)	C17—C18	1.378 (2)
C5—H5	0.967 (17)	C18—H18	0.945 (17)
C5—C6	1.391 (2)	C18—C19	1.398 (2)
C6—C7	1.493 (2)	C19—C20	1.436 (2)
C6—C11	1.395 (2)		
			
C3—O1—C2	116.50 (12)	C10—C9—H9b	112.9 (10)
C9—O3—C7	115.44 (11)	H10a—C10—C9	109.7 (11)
C14—O5—C12	116.74 (11)	H10b—C10—C9	110.6 (11)
H1b—C1—H1a	108.8 (16)	H10b—C10—H10a	109.5 (15)
H1c—C1—H1a	108.1 (16)	H10c—C10—C9	108.3 (12)
H1c—C1—H1b	107.9 (17)	H10c—C10—H10a	110.4 (16)
C2—C1—H1a	110.9 (11)	H10c—C10—H10b	108.3 (16)
C2—C1—H1b	110.5 (12)	H11—C11—C6	121.8 (10)
C2—C1—H1c	110.5 (12)	C12—C11—C6	118.62 (14)
C1—C2—O1	110.56 (13)	C12—C11—H11	119.5 (10)
H2a—C2—O1	108.7 (10)	C11—C12—O5	119.28 (13)
H2a—C2—C1	111.7 (11)	C13—C12—O5	118.64 (13)
H2b—C2—O1	103.5 (11)	C13—C12—C11	122.08 (13)
H2b—C2—C1	111.8 (11)	C12—C13—C4	118.85 (14)
H2b—C2—H2a	110.3 (16)	H13—C13—C4	120.5 (10)
O2—C3—O1	124.32 (14)	H13—C13—C12	120.7 (10)
C4—C3—O1	112.32 (12)	C15—C14—O5	124.64 (13)
C4—C3—O2	123.35 (14)	C19—C14—O5	115.53 (13)
C5—C4—C3	122.10 (13)	C19—C14—C15	119.83 (14)
C13—C4—C3	117.47 (13)	H15—C15—C14	119.3 (11)
C13—C4—C5	120.30 (14)	C16—C15—C14	119.51 (14)
H5—C5—C4	120.5 (10)	C16—C15—H15	121.2 (11)
C6—C5—C4	119.60 (13)	H16—C16—C15	118.6 (10)
C6—C5—H5	119.9 (10)	C17—C16—C15	121.22 (15)
C7—C6—C5	122.14 (13)	C17—C16—H16	120.2 (10)
C11—C6—C5	120.52 (14)	H17—C17—C16	120.5 (11)
C11—C6—C7	117.28 (13)	C18—C17—C16	119.28 (15)
O4—C7—O3	124.15 (13)	C18—C17—H17	120.3 (11)
C6—C7—O3	112.38 (12)	H18—C18—C17	119.8 (10)
C6—C7—O4	123.46 (13)	C19—C18—C17	120.36 (14)
H9a—C9—O3	107.7 (10)	C19—C18—H18	119.8 (10)
H9b—C9—O3	107.3 (10)	C18—C19—C14	119.78 (14)
H9b—C9—H9a	109.9 (14)	C20—C19—C14	119.90 (13)
C10—C9—O3	106.54 (13)	C20—C19—C18	120.31 (13)
C10—C9—H9a	112.2 (10)	C19—C20—N1	178.46 (16)

### Hydrogen-bond geometry (Å, º)

*Cg*1 is the centroid of the C4–C6/C11–C13 ring.

**Table:**

*D*—H···*A*	*D*—H	H···*A*	*D*···*A*	*D*—H···*A*
C2—H2B···O4^i^	0.98 (2)	2.50 (2)	3.2071 (2)	128 (1)
C13—H13···O4^ii^	0.936 (16)	2.514 (16)	3.4179 (2)	163.5 (12)
C16—H16···O2^iii^	0.997 (17)	2.516 (17)	3.1941 (2)	125.7 (13)
C18—H18···N1^iv^	0.945 (17)	2.629 (17)	3.430 (2)	143.0 (13)
C10—H10C···*Cg*1^v^	1.00 (2)	2.96 (2)	3.7893 (19)	140 (2)

Symmetry codes: (i) *x*, −*y*+3/2, *z*−1/2; (ii) −*x*+1, *y*−1/2, −*z*+3/2; (iii) *x*, −*y*+1/2, *z*+1/2; (iv) −*x*+2, −*y*+1, −*z*+2; (v) −*x*, *y*+1/2, −*z*+3/2.
